# A chromosome-level genome assembly of *Neotoxoptera formosana* (Takahashi, 1921) (Hemiptera: Aphididae)

**DOI:** 10.1093/g3journal/jkac164

**Published:** 2022-07-01

**Authors:** Shuai Ye, Chen Zeng, Jian-Feng Liu, Chen Wu, Yan-Fei Song, Yao-Guo Qin, Mao-Fa Yang

**Affiliations:** Institute of Entomology, Guizhou University; Guizhou Provincial Key Laboratory for Agricultural Pest Management of the Mountainous Region; Scientific Observing and Experimental Station of Crop Pest in Guiyang, Ministry of Agriculture, Guiyang, 550025, China; Puding Plant Protection and Quarantine Station, Guizhou, Puding 562100, China; Institute of Entomology, Guizhou University; Guizhou Provincial Key Laboratory for Agricultural Pest Management of the Mountainous Region; Scientific Observing and Experimental Station of Crop Pest in Guiyang, Ministry of Agriculture, Guiyang, 550025, China; The New Zealand Institute for Plant and Food Research Limited, Auckland 1142, New Zealand; Institute of Entomology, Guizhou University; Guizhou Provincial Key Laboratory for Agricultural Pest Management of the Mountainous Region; Scientific Observing and Experimental Station of Crop Pest in Guiyang, Ministry of Agriculture, Guiyang, 550025, China; Department of Entomology and MOA Key Laboratory for Monitoring and Environment-Friendly Control of Crop Pests, College of Plant Protection, China Agricultural University, Beijing 100193, China; Institute of Entomology, Guizhou University; Guizhou Provincial Key Laboratory for Agricultural Pest Management of the Mountainous Region; Scientific Observing and Experimental Station of Crop Pest in Guiyang, Ministry of Agriculture, Guiyang, 550025, China; College of Tobacco Science, Guizhou University, Guiyang 550025, China

**Keywords:** *Neotoxoptera formosana*, genome assembly, chromosome-level genome, gene family evolution, Hi-C

## Abstract

*Neotoxoptera formosana* (Takahashi), the onion aphid, is an oligophagous pest that mainly feeds on plants from the *Allium* genus. It sucks nutrients from the plants and indirectly acts as a vector for plant viruses. This aphid causes severe economic losses to *Allium tuberosum* agriculture in China. To better understand the host plant specificity of *N. formosana* on *Allium* plants and provide essential information for the control of this pest, we generated the entire genome using Pacific Biosciences long-read sequencing and Hi-C data. Six chromosomes were assembled to give a final size of 372.470 Mb, with an N50 scaffold of 66.911 Mb. The final draft genome assembly, from 192 Gb of raw data, was approximately 371.791 Mb in size, with an N50 contig of 24.99 Kb and an N50 scaffold of 2.637 Mb. The average GC content was 30.96%. We identified 73 Mb (31.22%) of repetitive sequences, 14,175 protein-coding genes, and 719 noncoding RNAs. The phylogenetic analysis showed that *N. formosana* and *Pentalonia nigronervosa* are sister groups. We found significantly expanded gene families that were involved in the THAP domain, the DDE superfamily endonuclease, zinc finger, immunity (ankyrin repeats), digestive enzyme (serine carboxypeptidase) and chemosensory receptor. This genome assembly could provide a solid foundation for future studies on the host specificity of *N. formosana* and pesticide-resistant aphid management.

**Significance**

Onion aphids cause significant economic losses to *Allium* plant agriculture, particularly *A. tuberosum*. However, there is very little knowledge of this aphid, in terms of genetics. To expand the genetic resource of this pest, we assembled the whole genome and found the expanding gene families of *N. formosana*. This will provide opportunities for future studies on *N. formosana* genetics and will eventually contribute to aphid control.

## Introduction

*Allium* crop species are used worldwide as vegetables and play an important role in daily diets in Asia (Shahrajabian *et al.* 2020). These *Allium* vegetables contain various sulfur and organic compounds that exhibit anticancer activities and may be useful for the treatment and prevention of cancers (Asemani *et al.* 2019). During our previous investigation, we found that *Neotoxoptera formosana* (Takahashi) (Hemiptera: Aphididae) causes significant economic losses to *Allium* plant agriculture, particularly *A. tuberosum*. *N.* *formosana*, also known as the onion aphid, damages *Allium* plants by sucking the cell sap from the plants, spreading many plant viruses and defecating sticky honeydew (Wang *et al.* 2021; Lin *et al.* 2022).

Onion aphids can search for *Allium* plants but show a significant response to the repellent effects of volatile sulfides, which are released by the plants (Hori 2007), and substances such as rosemary (Hori and Komatsu 1997). This aphid survives all year round but there are 2 main hazard peaks in the year: March–May and July–September, in Guizhou province, China. Previous studies have found that the predatory gall midge, *Aphidoletes aphidimyza*, has a good control efficiency against *N. formosana* under laboratory conditions (Wang *et al.* 2021). The complete mitochondrial genome of *N. formosana* was sequenced and annotated by Song *et al.* (2021). Despite its highly specialized host range and significant economic losses to *Allium* plant agriculture, no genome information for *N. formosana* has been published. In this study, we provided a chromosome-level genome assembly of *N. formosana*. This is the first genome assembly of this genus and will provide important basic information for the study of aphid taxonomy, host plant specificity, and pesticide-resistant aphid management.

## Materials and methods

### Sample collection and sequencing

**Fig. 1.**
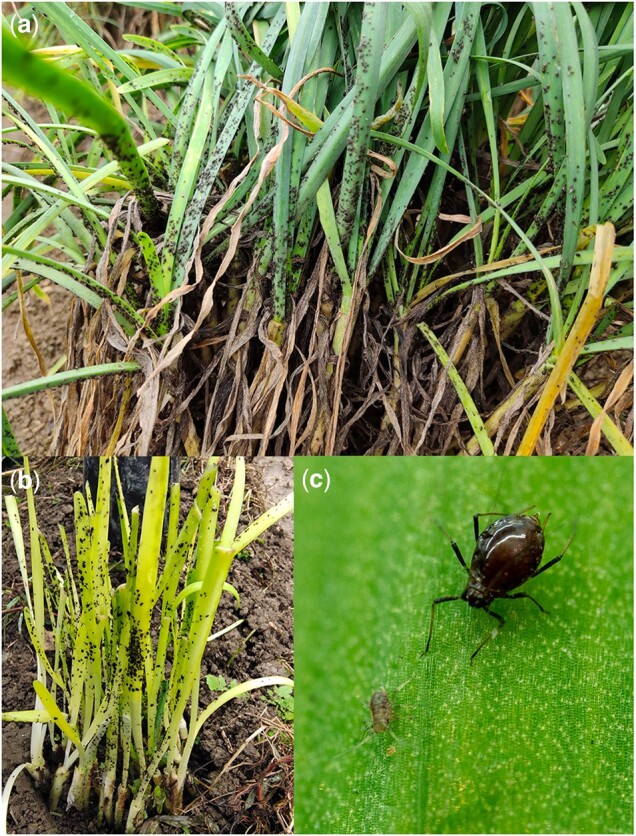
*Neotoxoptera formosana*. a) *N. formosana* damaging *Allium tuberosum*; b) *N. formosana* damaging yellow *A. tuberosum*; c) *N. formosana* female and nymph.

The onion aphids used for sequencing were obtained from Puding County, Anshun City, Guizhou Province, China (105˚ 27ʹ 49″ E, 26˚ 26ʹ 36″ N), in December 2020, and were reared in the laboratory at the Institute of Entomology, Guizhou University (Fig. 1). There were 90, 30, and 60 adult females used for PacBio sequencing, RNA-Seq analysis, and Hi-C sequencing, respectively. High-quality DNA was extracted using the QIAGEN DNeasy Blood and Tissue kit. For PacBio sequencing, a 20-kb insert-size library was constructed using the SMRTbell Template Prep Kit 2.0 and sequencing was performed on the PacBio Sequencer Sequel II. For the Illumina sequencing, the Truseq DNA PCR-free kit was used to construct PCR-free libraries with an insert size of 350 bp. For the RNA-Seq analysis, the TRIzol Reagent kit was used to extract RNA and libraries were constructed using the TruSeq RNA v2 kit. The Hi-C library construction was performed by Berry Genomics and included cross-linking, restriction enzyme (MboI) digestion, fragment end repair, DNA cyclization, and DNA purification. All the Illumina libraries were sequenced on a NovaSeq 6000, to achieve reads of 150 bp in length.

### Genome assembly

The Illumina genomic datasets were assessed for quality and trimmed using BBTools v38.82 (Bushnell 2014), using the following steps: (1) the duplicated sequences were removed using clumpify.sh; (2) bbduk.sh was used to remove low quality bases (<Q20), sequences shorter than 15 bases and poly-A/G/C tails (longer than 10 bases). It was also used to correct bases from overlapping reads (qtrim = rl, trimq = 20, minlen = 15, ecco = t, maxns = 5, trimpolya = 10, trimpolyg = 10, trimpolyc = 10). The k-mer analysis was performed using Genomescope v2.0 (Vurture *et al.* 2017), with the maximum k-mer coverage of 10,000. The kmer frequency was calculated using the khist.sh script from BBTools (kmer length: 21). The PacBio raw long reads that passed quality control were assembled using wtdbg2 v2.5 (Ruan and Li 2020), with the parameters of “-X 300 -p 15 -k 0 -S 4 -e 2.” Polishing of the assembly was performed using NextPolish v1.3.1 (Hu *et al.* 2020), with 1 round of long-read polishing and 2 rounds of short-read polishing. For short-read polishing, the reads were first mapped to the assembly using minimap2 v2.22 (Li 2018), with default parameters, and the produced “.sam” files were converted to “.bam” using samtools v1.10 (Li *et al.* 2009). The haplotypic duplications were removed from the assembly using Purge_dups v1.2.5 (Guan *et al.* 2020), with “-a 70.” To assign contigs to chromosomes, Juicer v1.6.2 (Durand *et al.* 2016) was first used to align the high-quality Hi-C reads to the assembly and the contigs were then scaffolded using 3D-DNA v180922 (Dudchenko *et al.* 2017), with default parameters. The generated pre-pseudochromosomes were manually corrected using Juicerbox v1.11.08 (Durand *et al.* 2016), based on the Hi-C contact maps, and the files were then imported into 3D-DNA to produce the final chromosomal assembly. The contaminated sequences were assessed and removed, using MMseqs2 v12-113e3 (Steinegger and Söding 2017), by blasting contigs against the *nt* and UniVec databases. The cleaned assembly was also uploaded to NCBI for an additional search for possible contamination. The assembly completeness was assessed using BUSCO v3.0.2 (Waterhouse *et al.* 2018), with searches against “insect_odb10.” To assess the coverage of raw data, the reads were mapped to the assembly using Minimap2 v2.22. To investigate genome collinearity with *Acyrthosiphon pisum* and *Rhopalosiphum maidis*, MMseq2 v12-113e3 was first used to align protein sequences with “blastp,” with parameters of “s 7.5 –alignment-mode 3 –num-iterations 4 -e 1e-5 –max-accept 5.” The resulting files, together with the annotation file (all.gff), were then used as inputs to MCScanX for collinearity analysis and were visualized using TBtools v1.0692 (Chen *et al.* 2020).

### Genome annotation

We annotated repeats, protein-coding genes, and noncoding RNAs from the assembly. To identify repeats, RepeatModeler v2.0.2a (Flynn *et al.* 2020) was first used to generate a de novo repeat library with the parameter of “-LTRStruct.” This repeat library was then merged with the sequences from the RepBase-20181026 (Bao *et al.* 2015) database to form a more extensive repeat library, which was used as the input into RepeatMasker v4.1.0 (Smit *et al.* 2013–2015). The protein-coding gene models were predicted using MAKER v3.01.03 (Holt and Yandell 2011), by integrating the predictions from 3 strategies. EVidenceModeler (EVM) was used for evidence weighting. The 3 strategies were: (1) ab initio prediction: BRAKER v2.1.6 (Hoff *et al.* 2016) was used to train Augustus v3.4.0 (Stanke *et al.* 2004) and GeneMark-ES/ET/EP 4.68_lic (Brůna *et al.* 2020) and then predict genes with the evidence from RNA-Seq data, to improve accuracy. Alignments from the RNA-Seq analysis were generated using HISAT2 v2.2.1 (Kim *et al.* 2019). (2) Transcript-based gene structure prediction: a reference-guided transcriptome assembly was generated using StringTie v2.1.6 (Kovaka *et al.* 2019), by assembling RNA-Seq reads. This assembly was then aligned to the genome assembly using HISAT2. (3) Protein-homology-based prediction: the characterized protein sequences from the phylogenetically close species, *Acyrthosiphon pisum*, *Drosophila melanogaster*, *Nilaparvata lugens*, *Thrips palmi*, *Rhopalosiphum maidis*, and *Pediculus humanus*, were downloaded from NCBI for the model.

Gene functional annotation was conducted in 3 steps: (1) gene models were searched against the UniProtKB (SwissProt+TrEMBL) and *nr* databases. To search against UniProtKB, the sensitive mode (–very-sensitive -e 1e-5) was used for Diamond v2.0.11.149 (Buchfink *et al.* 2015) to obtain functional description; (2) gene models were searched against the Pfam, Smart, Superfamily, and CDD databases using InterProScan 5.48-83.0 (Quevillon *et al.* 2005) and the eggNOG v5.0 (Huerta-Cepas *et al.* 2019) database, with eggNOG-mapper v2.1.5 (Huerta-Cepas *et al.* 2017). These data were used to predict protein domains, gene ontology (GO) terms, KEGG, and Reactome pathways. (3) The results generated from the above were integrated to produce the final functional annotation.

To annotate noncoding RNAs, infernal v1.1.4 was used to annotate rRNA, snRNA, and miRNA by searching against the Rfam database. The tRNAs were annotated using tRNAscan-SE v2.0.9 (Chan and Lowe 2019) and the predicted tRNAs with low fidelity were removed with the “EukHighConfidenceFilter” script.

### Species phylogeny and gene family evolution

We downloaded protein sequences from 15 species from NCBI to infer gene family homology. These species covered orders, tribes, and families and included *Thrips palmi*, *Acyrthosiphon pisum*, *Sitobion miscanthi*, *Diuraphis noxia*, *Myzus persicae*, *Aphis gossypii*, *Melanaphis sacchari, Pentalonia nigronervosa*, *Rhopalosiphum maidis*, *Eriosoma lanigerum*, *Sipha flava*, *Nilaparvata lugens*, *Phenacoccus solenopsis*, *Riptortus pedestris*, and *Pachypsylla venusta*. The sequences were initially clustered using OrthoFinder v2.3.8 (Emms and Kelly 2019) and then aligned using Diamond. To generate alignments for species phylogenetic tree construction, the 1,273 single-copy gene clusters were aligned individually to generate homologous regions using MAFFT v7.453 (Katoh and Standley 2013), with “L-INS-I.” The regions that were inappropriately aligned were trimmed using trimAl v1.4.1. To construct the phylogeny, FASconCAT-G v1.04 (Kück and Longo 2014) was used to generate a supermatrix as the input for IQ-TREE v2.1.3 (Minh *et al.* 2020), with settings of “–symtest-remove-bad –symtest-pval 0.10 –m MFP –mset LG –msub nuclear –rclusterf 10 –B 1000 –alrt 1000.”

The estimation of evolutionary time because species divergence was performed using MCMCTREE, from the PAML v4.9j package, with parameters of “clock = 2, RootAge = < 3.827, model = 0, BDparas = 1 1 0.1, kappa_gamma = 6 2, alpha_gamma = 1 1, rgene_gamma = 2 20 1, sigma2_gamma = 1 10 1.” There were 6 sets of fossil evidence downloaded from the PBDB database (https://www.paleobiodb.org/navigator/) and used as calibrations for this estimation: Hemiptera and Thysanoptera (<3.827 MYA) as the root, Sternorrhyncha (3.146–3.589 MYA), Aphalaridae and Pseudococcidae (2.793–3.232 MYA), Aphididae (0.996–1.4 MYA), Macrosiphini (>0.339 MYA), and *Nilaparvata lugens* and *Riptortus pedestris* (2.989–3.232 MYA).

The prediction of gene family expansion and contraction within *N. formosana*, when compared with the other 15 species, was conducted using CAFÉ v4.2.1, with the model of single birth-death parameter lambda and a significance level of 0.01 (*P* = 0.01). The significantly expanded/contracted gene families were then assigned to GO and KEGG categories, using R package clusterProfiler v3.10.1 (Yu *et al.* 2012), with the default parameters (*P* = 0.01 and *q* = 0.05).

## Results and discussion

### Genome sequencing and assembly

**Fig. 2.**
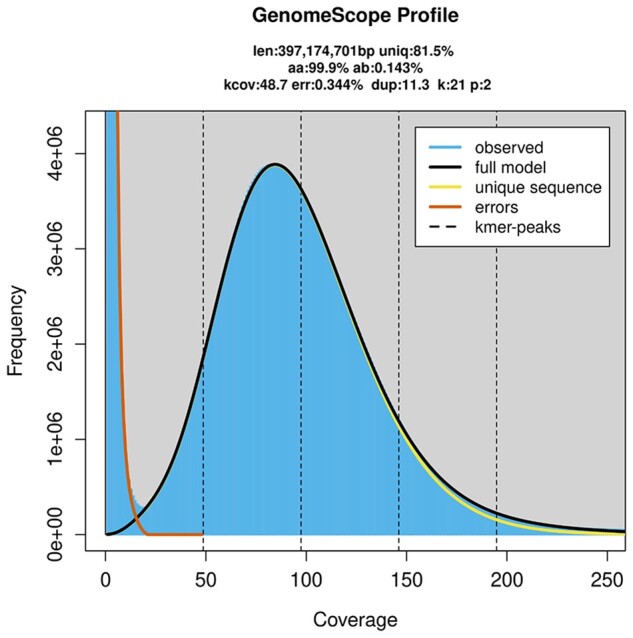
The K-mer frequency distribution analysis of *Neotoxoptera formosana*.

**Fig. 3.**
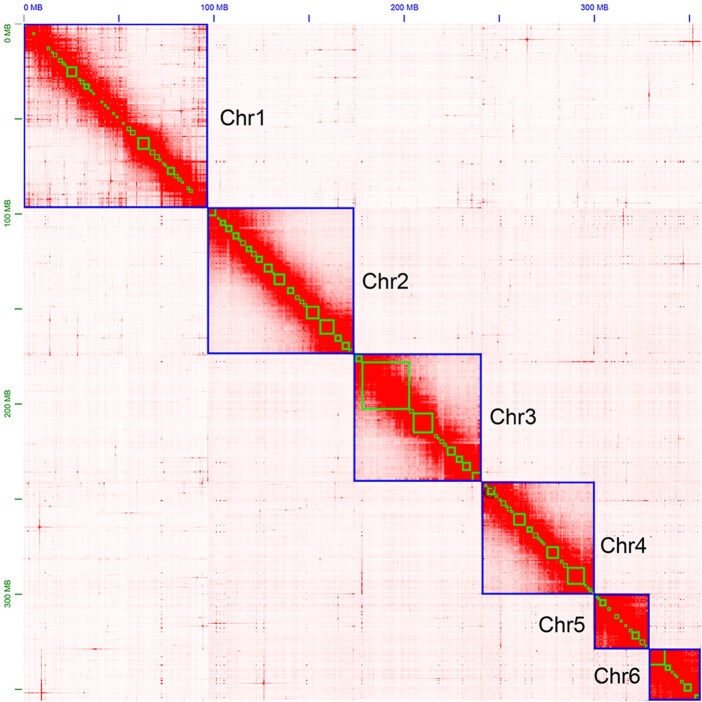
Hi-C contact map of the *Neotoxoptera formosana* genome.

**Fig. 4.**
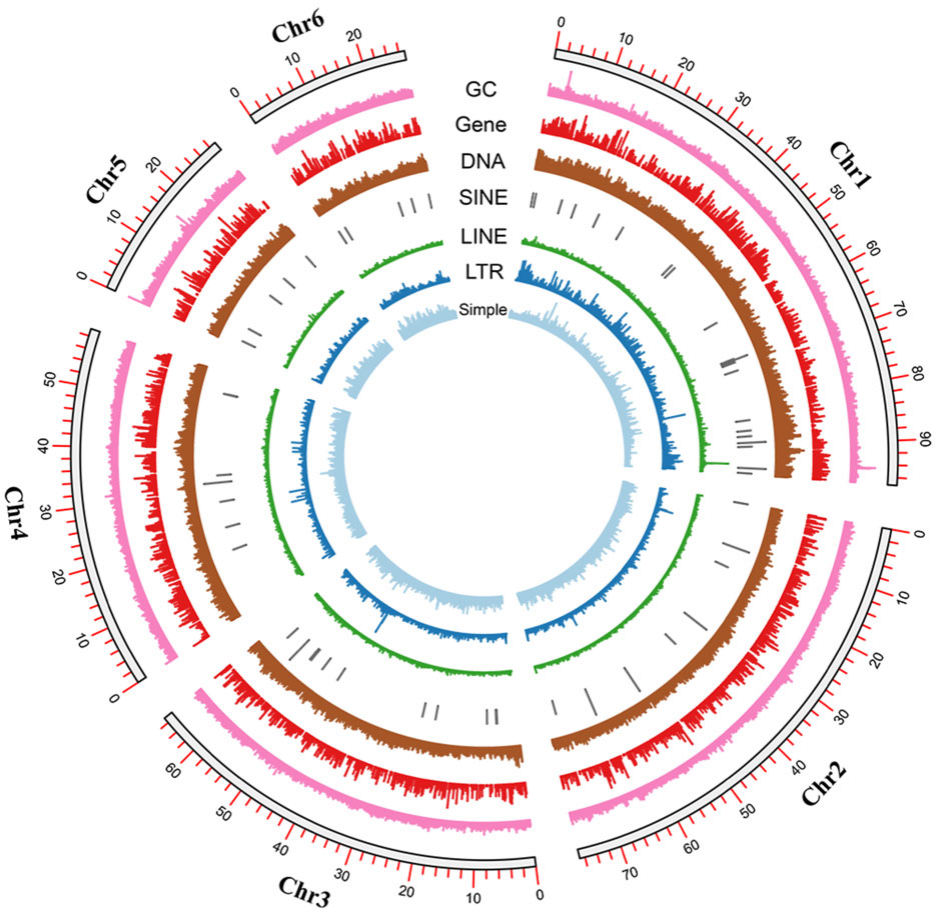
Circos plot that indicates chromosome length, GC content, and protein-coding gene/repeat sequence density.

We obtained 104.3 Gb of PacBio long reads, with a mean read length of 15.42 kb and an N50 length of 24.99 kb. The genome was predicted to be between 395.5 and 397.2 Mb, with extremely low heterozygosity (Fig. 2). We estimated that 31.22% of the assembly contained regions of repetitive sequences (Supplementary Table 1). Our initial genome assembly, which was assembled solely from PacBio reads, was 371.791 Mb and contained 357 scaffolds and 1259 contigs (Table 1). We found that 93.9% of this assembly contained complete BUSCO genes (1,367), with a duplicate gene rate of 2.3% (Table 2). The mapping-back rates for Illumina short and PacBio long reads were 96.79% and 92.95%, respectively, which indicated that our assembly had high coverage of the raw data. There were approximately 800 Mb of Hi-C data used to assign scaffolds and contigs onto the 6 chromosomes (Figs. 3 and 4).

**Table 1.** Genome assembly statistics of *Neotoxoptera formosana.*

Assembly	Total length (Mb)	Number of scaffolds	N50 length (Mb)	Longest scaffold (Mb)	GC (%)	BUSCO (*n* = 1,367) (%)			
						C	D	F	M
wtdbg	393.678	2,806	2.637	24.999	31.55	92.1	2.6	2.0	5.9
NextPolish	392.017	2,806	2.632	24.933	31.59	94.0	2.6	0.9	5.1
Purge_dups	376.840	1,911	2.800	24.933	31.15	94.0	2.6	0.9	5.1
3D-DNA	372.470	461	66.911	97.256	30.98	93.9	2.3	0.9	5.2
Final	371.791	357	66.908	97.223	30.96	93.9	2.3	0.9	5.2

**Table 2.** Genome assembly and annotation statistics for *Neotoxoptera formosana*.

	*Neotoxoptera formosana*
Genome assembly	
Assembly size (Mb)	371.791
Number of scaffolds/contigs	357/1,259
Longest scaffold/contig (Mb)	97.223/24.933
N50 scaffold/contig length (Mb)	66.908/2.772
GC (%)	30.96
Gaps (%)	0.024
BUSCO completeness (%)	93.9%
Gene annotation	
Protein-coding genes	14,175
Mean protein length (aa)	504.5
Mean gene length (bp)	6,552.6
Exons/introns per gene	9.3/8.0
Exon (%)	9.64
Mean exon length	271.4
Intron (%)	15.34
Mean intron length	501.4
BUSCO completeness (%)	93.9

### Genome annotation

We annotated the repetitive sequences, protein-coding genes, and noncoding RNAs from the genome assembly. There were 754,839 predicted repetitive sequences, which made-up approximately 116 Mb (31.22%) of the assembly. The 5 most abundant repeat types were DNA elements (11.64%), unclassified (10.72%), simple repeats (4.09%), LINEs (1.92%), and LTR elements (1.21%) (Supplementary Table 1). There were 14,175 predicted protein-coding genes, supported by approximately 8 Gb of RNA-Seq data. The predicted genes had a mean length of 6,552.6 bp, a mean CDS length of 211.1 bp and a mean number of exons of 9.3 (Table 1). Of the genes that were completely recovered from this gene set, 93.9% were BUSCO genes. InterProScan identified protein domains for 11,227 predicted protein-coding genes and, together with eggNOG results, 9,607 and 8,287 genes were annotated with gene ontology (GO) terms and KEGG pathways, respectively.

We annotated 719 noncoding RNAs that contained 128 miRNAs, 89 rRNAs, 97 snRNAs, 225 tRNAs, 27 ribozymes, 4 lncRNAs, and 149 other RNAs. The 97 snRNAs included 47 G4-forming RNAs (U1, U2, U4, U5, U6, and U11), 3 minor G4-forming RNAs (U4atac, U6atac, and U12) and 47 C/D box snoRNAs (Supplementary Table 2).

### Genome collinearity analysis

**Fig. 5.**
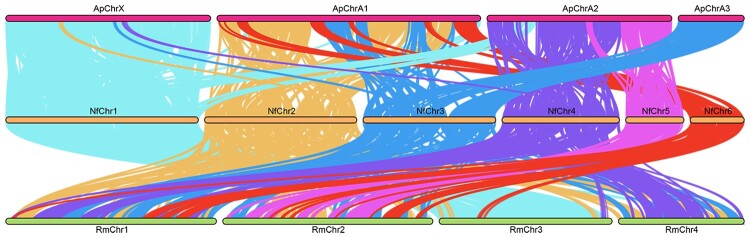
Chromosome collinearity analysis graph. Ap: *Acyrthosiphon pisum*; Nf: *Neotoxoptera formosana*; Rm: *Rhopalosiphum maidis*.

We found a relatively high level of conserved linkage between *N. formosana* (Nf) and *Acyrthosiphon pisum* (Ap) genomes, when compared with Nf and *Rhopalosiphum maidis* (Rm) genomes (Fig. 5). *N.* *formosana* chromosome 1 (NfChr1) was mostly collinear with ApChrX, with some small regions aligned to ApChrA2. We found that NfChr2 and NfChr6 had homologous regions on ApChrA1. The syntenic regions of NfChr3 were located on ApChrA1 and the entire of ApChrA3. Conservation was observed between NfChr4 and 5 and ApChrA2. When compared with the *R. maidis* genome, only NfChr1 showed a high level of conservation with RmChr3, whilst other chromosomes had syntenic regions scattered across the *R. maidis* genome.

### Phylogeny

**Fig. 6.**
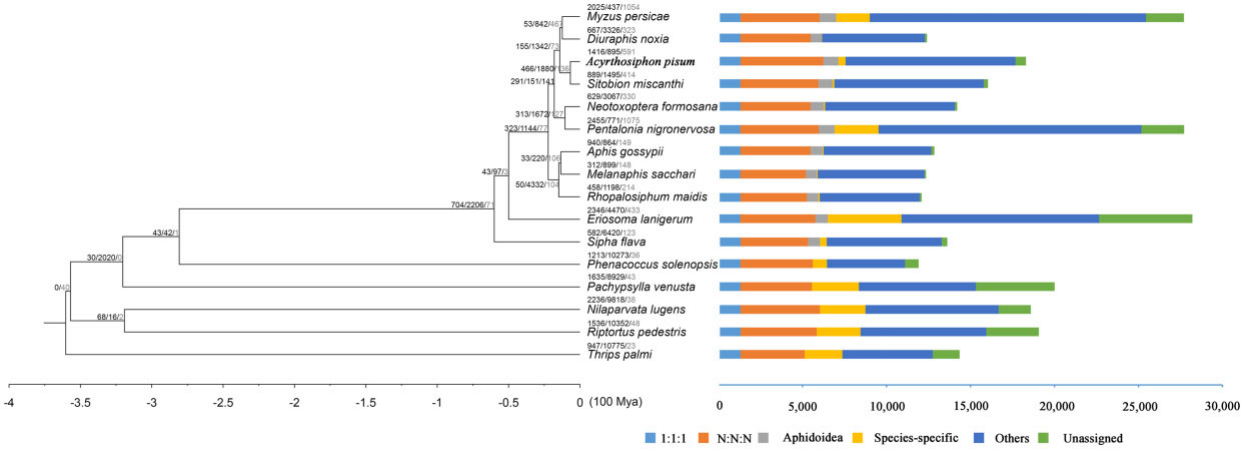
Phylogenetic tree of *Neotoxoptera formosana*: The branch length represents evolution time, numbers represent the number of expanded, contracted and rapidly evolving (statistically significant, labeled as red) gene families in that branch.

We used protein sequences from 15 species, together with the annotated *N. formosana* protein models, to construct a phylogenetic tree (Fig. 6). There were 254,609 (91.3%) gene models assigned to 19,010 gene families. Among 4,169 gene families that were present in all species, 1,273 and 2,896 were single- and multi-copy families, respectively. In *N. formosana*, 14,037 genes were clustered into 9,838 families and 77 genes from 30 families were found to be specific to this species (Fig. 6). A total of 555,217 amino acid residues, obtained from 1,113 single-copy genes, were used for phylogenetic construction. Most lineages had UFB/SH-aLRT supports of 100/100, apart from *Macrosiphini sacchari*, which had supports of 99.9/94, and *Aphis gossypii* and *Melanaphis sacchar*, which had supports of 99.3/98 (Fig. 6). This phylogeny suggested that *N. formosana* and *P.* *nigronervosa* were sister groups.

### Gene family evolution

**Fig. 7.**
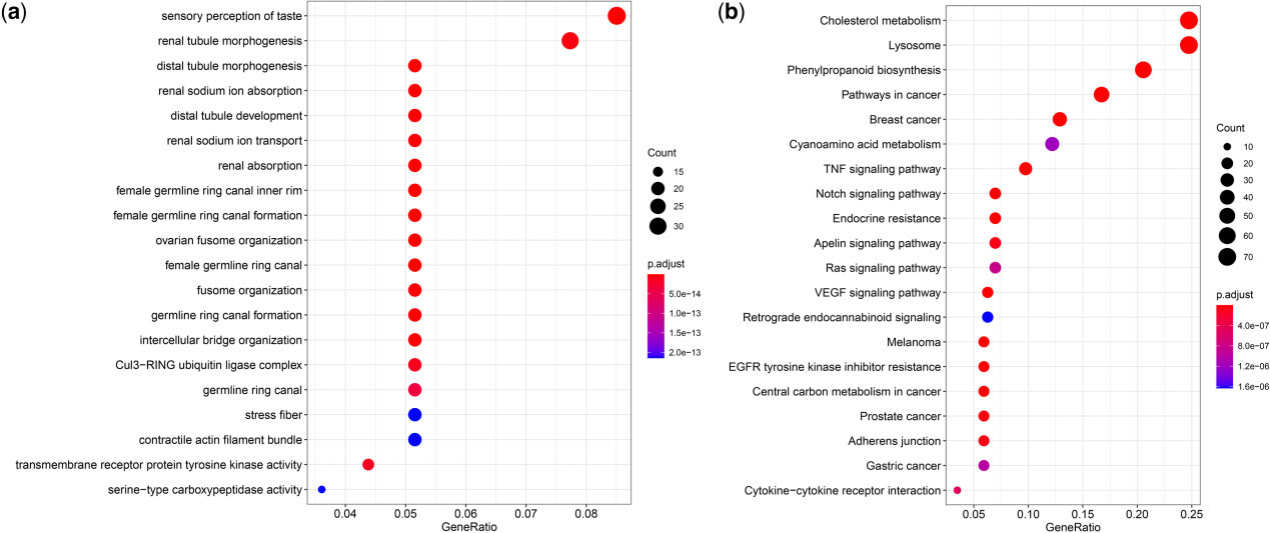
Gene family evolution of *Neotoxoptera formosana*. a) Bubble plot of GO enrichment analysis of rapidly expanding gene families; b) bubble plot of KEGG enrichment analysis of rapidly expanding gene families.

When compared with the other species, we found that the number of expanded and contracted gene families in *N. formosana* was 629 and 3,067, respectively. There were 330 gene families that showed significant expansion or contraction. The gene families with significant expansion were THAP domain, DDE superfamily endonuclease, zinc finger, immunity (ankyrin repeats), digestive enzyme (serine carboxypeptidase), and chemosensory receptor families (Fig. 7a). The Odorant receptors (ORs) gene family exhibited rapid expansion, in accordance with the GO enrichment analysis. The results of the KEGG pathway enrichment analysis showed that pathways involved in detoxification, immunity, and secondary metabolite synthesis were significantly enriched (Fig. 7b).

Aphid ORs play an essential role in the perception of different host odors or pheromones (Liu *et al.* 2022). In this study, 16 ORs candidate genes were rapidly exhibited expansion in *N. formosana*. This rapid expansion might be associated with the feeding behaviour of *N. formosana*, which is an oligophagic aphid pest only feeding on different *Allium* species. Hori (2007) finds that *N. formosana* might use dipropyl trisulphide (extracted from *Allium fistulosu*) and diallyl disulphide (extracted from *Allium tuberosum*) as olfactory cues to search for the host plants based on Y-tube olfactometer. In order to understand the odor perception of *N. formosana*, future work should analyze the expression patterns of ORs genes in different issues and identify the functional analysis of ORs genes to different plant volatiles (Zhang *et al.* 2019).

## Data availability

Genome assembly and raw sequencing data have been deposited at the NCBI under the accessions JAIWJD000000000 and SRR18085628, SRR18079676, SRR18079766 and SRR13334673, respectively. Genome annotations are available at the Figshare under the link: https://figshare.com/articles/online_resource/A_Chromosome-level_genome_assembly_of_Neotoxoptera_formosana_Takahashi_Takahashi_1921_Hemiptera_Aphididae_/19165817.

Supplemental material is available at *G3* online.

## Supplementary Material

jkac164_Supplementary_Data
